# Draft Genome Sequence of Muricauda sp. Strain K001 Isolated from a Marine Cyanobacterial Culture

**DOI:** 10.1128/genomeA.00451-18

**Published:** 2018-05-31

**Authors:** Carla S. Vizzotto, Fabyano A. C. Lopes, Stefan J. Green, Andrei S. Steindorff, Juline M. Walter, Fabiano L. Thompson, Ricardo H. Krüger

**Affiliations:** a Department of Cellular Biology, Biological Sciences Institute, University of Brasília, Brasília, Brazil; b Department of Civil and Environmental Engineering, University of Brasília, Brasília, Brazil; c DNA Services Facility, Research Resources Center, University of Illinois at Chicago, Chicago, Illinois, USA; d Laboratory of Microbiology, Institute of Biology, Federal University of Rio de Janeiro, Rio de Janeiro, Brazil; e Center of Technology—CT2, SAGE-COPPE, Federal University of Rio de Janeiro, Rio de Janeiro, Brazil

## Abstract

We report the whole-genome sequence of Muricauda sp. strain K001 isolated from a marine cyanobacterial culture. This genome sequence will improve our understanding of the influence of heterotrophic bacteria on the physiology of cyanobacteria and may contribute to the development of new natural products.

## GENOME ANNOUNCEMENT

The heterotrophic bacterium Muricauda sp. strain K001 was isolated from marine cyanobacterial culture CCMR0080 (Culture Collection of Microorganisms at the Federal University of Rio de Janeiro). Muricauda spp. are rod-shaped Gram-negative bacteria widely distributed in seawater around the world. They form small yellow colonies with regular edges and are able to synthetize zeaxanthin (1–3). Although the association between cyanobacteria and heterotrophic bacteria in several environments and in culture has been described (4–6), this relationship is not well understood. Members of the genus Muricauda have been reported to be associated with filamentous cyanobacteria in laboratory culture (7). To date, however, there has been no whole-genome sequence (WGS) of a Muricauda strain grown in coculture with any other organism. This study presents the first WGS for Muricauda isolated from cyanobacterial culture.

Muricauda sp. strain K001 was cultured in Marine broth 2216 (Difco) with agar for 36 h at 30°C. Genomic DNA was extracted from a single colony using the GenElute bacterial genomic DNA kit (catalog no. NA2110, Sigma-Aldrich), according to the manufacturer’s instructions. A library for sequencing was created using the Nextera XT kit (Illumina, San Diego, CA), according to the manufacturer’s instructions. Sequencing was performed using an Illumina NextSeq500 platform with paired-end reads (read length, 150 bp). Raw sequence data were quality trimmed, and *de novo* assembly was performed in the software package CLC Genomics Workbench (Qiagen, Hilden, Germany). Sequencing and *de novo* assembly were performed in the DNA Services (DNAS) facility at the University of Illinois at Chicago. Complementary metric information was obtained with Quast version 4.6.3 (8). Prokka version 1.12 was used to annotate the genome using the UniProt database (9). Secondary metabolites were identified using antiSMASH (online version) (10). BUSCO version 3.0.2 was used to find single-copy orthologs (11).

The draft genome of Muricauda sp. strain K001 is approximately 3.8 Mb, distributed in 55 contigs, with an overall 41.6% G+C content. The average coverage was approximately 300×, and the *N*_50_ and *L*_50_ values for the assembly were 232,816 bp and 5 contigs, respectively. The annotation identified 3,408 genes, 3,356 coding sequences, 3 rRNAs, 35 transfer RNAs, 13 miscellaneous RNAs, 1 transfer-messenger RNA, and 424 signal peptides. The 16S rRNA gene sequence of Muricauda sp. strain K001 showed 98% identity with that of Muricauda ruestringensis strain DSM 13258 (GenBank accession no. NR_074562). The completeness score of the genome was 98.6% (437 complete single-copy orthologs from the 443 Bacteroidetes ortholog data set). Muricauda sp. strain K001 showed 20 gene clusters of secondary metabolites, with one cluster identified as being involved in carotenoid synthesis (34% similarity with M. ruestringensis strain DSM 13258).

This genome will improve our knowledge of the relationship between heterotrophic bacteria and primary producers by identifying the capabilities of the heterotrophic partner. In addition, this research may contribute to the development of valuable natural products, such as pigments and other biotechnologically important secondary metabolites.

### Accession number(s).

This whole-genome shotgun project has been deposited in DDBJ/ENA/GenBank under the accession no. QBTW00000000. The version described in this paper is the first version, QBTW01000000.
